# Mutation Rates, Mutation Frequencies, and Proofreading-Repair Activities in RNA Virus Genetics

**DOI:** 10.3390/v13091882

**Published:** 2021-09-21

**Authors:** Esteban Domingo, Carlos García-Crespo, Rebeca Lobo-Vega, Celia Perales

**Affiliations:** 1 Centro de Biología Molecular “Severo Ochoa” (CSIC-UAM), Consejo Superior de Investigaciones Científicas (CSIC), Campus de Cantoblanco, 28049 Madrid, Spain; carlos.garciac@cbm.csic.es; 2 Centro de Investigación Biomédica en Red de Enfermedades Hepáticas y Digestivas (CIBERehd), Instituto de Salud Carlos III, 28029 Madrid, Spain; 3 Department of Clinical Microbiology, Instituto de Investigación Sanitaria-Fundación Jiménez Díaz University Hospital, Universidad Autónoma de Madrid (IIS-FJD, UAM), Av. Reyes Católicos 2, 28040 Madrid, Spain; rebelobo@ucm.es

**Keywords:** RNA virus, virus diversification, quasispecies, error catastrophe, exonuclease, SARS-CoV-2

## Abstract

The error rate displayed during template copying to produce viral RNA progeny is a biologically relevant parameter of the replication complexes of viruses. It has consequences for virus–host interactions, and it represents the first step in the diversification of viruses in nature. Measurements during infections and with purified viral polymerases indicate that mutation rates for RNA viruses are in the range of 10^−3^ to 10^−6^ copying errors per nucleotide incorporated into the nascent RNA product. Although viruses are thought to exploit high error rates for adaptation to changing environments, some of them possess misincorporation correcting activities. One of them is a proofreading-repair 3′ to 5′ exonuclease present in coronaviruses that may decrease the error rate during replication. Here we review experimental evidence and models of information maintenance that explain why elevated mutation rates have been preserved during the evolution of RNA (and some DNA) viruses. The models also offer an interpretation of why error correction mechanisms have evolved to maintain the stability of genetic information carried out by large viral RNA genomes such as the coronaviruses.

## 1. Introduction: Mistakes as Hallmark of the Evolution of Life and of Present Day Viruses

Studies of prebiotic nucleotide synthesis suggest that the origin of the high mutation rates exhibited by many present day viruses may be attributed to a limited template-copied fidelity that likely operated during the early stages of life development. There are several models of life emergence, and all of them include at some stage replication of molecules that carried inheritable information. This statement is supported both by theoretical and experimental results that the reader can find in References [1,2,3,4,5,6]. According to such results, the evolution of primitive life forms was possible thanks to mistakes that unavoidably and blindly occurred during copying of the template molecules that carried the information to be transmitted. Production of error copies is inherent to subcellular and cellular life forms [1,2,3,4,5,6]. Several lines of evidence suggest that viruses have played a prominent role in the organization of cellular genomes, mainly as a result of their capacity to penetrate into cells and to transport, integrate, and exchange genetic material with that of cellular organizations under construction [5]. A number of mechanisms of integration of viral genomes (or part of them) into cellular genomes have been characterized in present day viruses. They include retroviral DNA integration, insertion of temperate viral DNA of lysogenic bacteria such as phage lambda into E. coli DNA, and bacterial DNA rescue by transducing viruses. These well-characterized integration events should be added to the extensive evidence of the presence of DNA copies of many pathogenic RNA viral genomes in their animal or insect hosts, in most cases by mechanisms that are not well understood [5].

Together with other mobile elements, viruses are thought to have promoted cellular variation and differentiation, often through co-evolutionary processes (reviewed in [5,7,8,9]; among other accounts). According to these studies, the perception of viruses as blind, selfish, and invasive replicators should be regarded only a facet of their natural history, and probably not the one responsible for their maintenance and abundance in the biosphere. To effectively participate in the construction of a cellular world, genome variation of the primitive replicons must have played a prominent role to cope with an increasing cellular differentiation and to overcome barriers to penetration into cells, as again evidenced by independent studies [5,8,9].

Many of the viruses that have been isolated and studied exhibit an “error-prone replication”, as they multiply in cells and organisms, in some cases with associated disease manifestations. From the studies summarized above [2,3,4,5,6,7,8,9,10], it has been proposed that this feature is probably a beneficial remnant of virus origins, combined with its usefulness for sustained adaptability [10,11,12]. Production of error copies underlies quasispecies dynamics (continuous production of variant genomes subjected to competition, selection, and random drift, and which may act as a unit of selection), which is crucial for virus adaptation to changing environments and for the collective behavior of viral populations [10,11,12,13].

Awareness of the medical consequences of mutations for viruses arose during the last century when viral genome analyses entered the scene as a tool to understand and confront viral diseases. Many studies focused on diseases that acquired epidemic and pandemic proportions, such as poliomyelitis, influenza, and AIDS. The ongoing COVID-19 pandemic represents the most recent scourge, with again prominence of genome variations of the causative virus.

During the massive vaccination campaigns to control poliovirus using the Sabin live-attenuated vaccine in the middle of last century, it was observed that approximately one out of a million vaccine recipients (or their contacts) developed paralytic disease. In the afflicted individuals, poliovirus had mutated to lose its attenuation phenotype. Vaccine-associated mutant and recombinant polioviruses emerged in different world locations. Poliovirus mutants and recombinants became a major problem for the global eradication of poliomyelitis which, to this date, is still incomplete due both to the adaptive capacity of poliovirus and to socio-political impediments [13,14,15]. 

For influenza, mutations that promote antigenic drift of influenza virus (IV) (the gradual accumulation of amino acid substitutions in the surface antigens hemagglutinin and neuraminidase) and genome segment reassortments that result in antigenic shift (exchange of genome segments that encode the surface antigens particularly between human and animal IVs) are behind periodic human pandemics. One of the consequences of antigenic drift and shift is the need to update the composition of the inactivated anti-influenza vaccines, which generally display a limited efficacy [16].

In the case of AIDS, the capacity of immunodeficiency virus type 1 (HIV-1) to escape components of the human immune response through mutation, the debilitated response due to the HIV-1 tropism for cells involved in the immune response, and the proviral DNA integration as part of the virus life cycle are reasons why to date there are no effective anti-AIDS vaccines [17,18]. Antiretroviral therapy has been successful in reducing AIDS and HIV-1 infection-related mortality, but antiretroviral resistance through genetic variation of the virus and selection of escape mutants remains an issue.

For COVID-19, we are confronting the seemingly paradoxical situation of a coronavirus that is supposed to limit its mutation rate via a proofreading-repair activity (Section 5) but that is continuously producing new variants that circulate in the human population and that represent a threat to vaccine efficacy [19,20,21]. 

The observations with four pandemic viruses, here summarized briefly, render unquestionable an essential role of genetic variation as effector of virus–host interactions and of difficulties for viral disease control. There are numerous additional examples of the relevance of mutation, recombination, and genome segment reassortment for viruses to overcome selective constraints. The latter are ubiquitous during all stages of a virus life cycle. Remarkably, they also include medical interventions, such as administration of antiviral agents, vaccines, or immunotherapy with polyclonal or monoclonal antibodies [5]. Genetic variation of viruses provides the key molecular mechanism for responding to selective constraints. Several ways of trying to counteract such virus capacities (without guaranteed success) have been proposed: use of multi-epitopic vaccines (ideally live-attenuated, with limited reversion potential), combination therapies, and new broad-spectrum antiviral designs such as lethal mutagenesis in synergistic combinations, among others [5,11,12]. The scope of implications of error-prone replication of RNA (and many DNA) viruses justifies focusing on the origin of mutations and mechanisms to limit their frequency. Here we review some studies on viral mutation rates and frequencies, as well as the evidence that, as for any replicative system, there is a limit to the number of mutations that can be accepted to preserve the encoded genetic information. Therefore, we also review why and how mutation rates may be modulated by proofreading-repair and post-replicative repair (error-correcting) activities.

## 2. Mutation Rates and Frequencies

**Figure 1:**
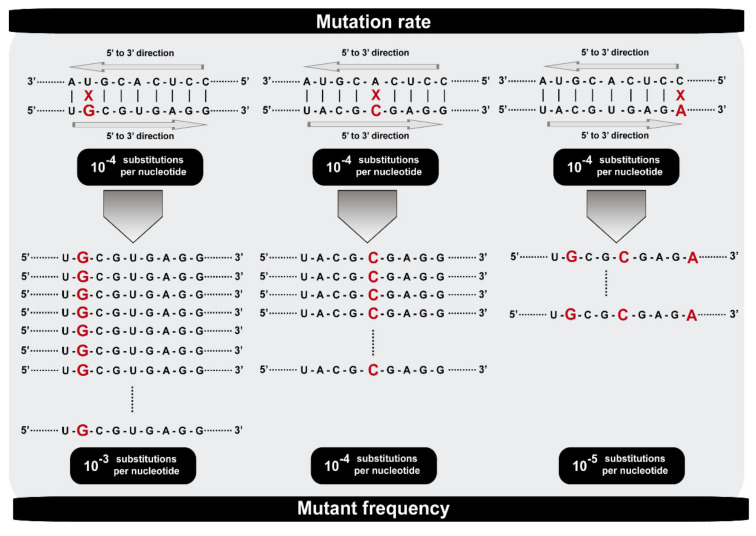
Scheme that illustrates the difference between mutation rate and mutant frequency. Residue U (on the left), A (in the middle), and C (on the right) can be misread to incorporate a G (on the left), C (in the middle), or A (on the right) in the complementary strand at a rate of 10^−4^ substitutions per nucleotide in all cases. The replicative capacity of the newly G, C, and A templates determines the different mutant frequencies with 10^−3^, 10^−4^, and 10^−5^ substitutions per nucleotide, respectively.

The term “mutation rate” is often used by geneticists as synonymous of “rate of evolution”. This indistinct use is misleading and hides relevant differences between these two parameters that describe evolutionary episodes that are only indirectly connected. “Mutation rate” should be used to mean the proportion of erroneous nucleotides introduced in a process of template copying to yield complementary strands and progeny viral genomes. It applies to events occurring during RNA or DNA synthesis in replication complexes. The newly arising mutations constitute the very first stage of virus diversification on which subsequent selective forces and random events act. “Rate of evolution” should be the term used to refer to the speed at which genomic sequences of viruses accumulate mutations in nature or in some experimental evolution setting as a function of time, leading to genome diversification. Within a replication complex, a specific mutation may arise at high rate (for example, under the influence of a particular sequence context in the template RNA) but be found at low frequency during subsequent rounds of intra-cellular template copying as a consequence of low replicative capacity (fitness). This gives rise to a third parameter termed “mutation frequency”. This parameter may be given for the average calculated for a set of mutations determined in a genome or genomic region, or for an individual mutation at a defined genomic site, in which case it is often termed a “mutant frequency”. The mutation frequency is dependent on (but not identical with) the mutation rate (Figure 1). Mutation frequencies dictate the repertoire of variant genomes that populate an infected host and that contribute the infectious particles that can be transmitted to other hosts.

A difference between a mutation rate and a mutation frequency is an example of conflict between a molecular instruction and a biological requirement, often encountered in the generation of mutant and recombinant genomes (reviewed in [5]). A pertinent example studied in our laboratory is the elongation of an internal oligoadenylate tract located between the two functional AUG triplets of the foot-and-mouth disease virus (FMDV) genome. The elongation was observed very frequently during plaque-to-plaque transfers of the virus in cell culture (an experimental design used to mimic the effect of repeated bottleneck events in viral populations), but it has never been observed in FMDV isolates from infected animals [22]. Fitness values of FMDV clones harboring the elongated oligoadenylate and quantification of the rate of reversion to its original length suggested that the site was a hot spot for homopolymeric tract elongation, likely through polymerase slippage (a molecular instruction). This genome modification was detectable probably because of the limited negative selection operating during plaque-to-plaque transfers, in contrast with the sieving effect (elimination of genomes harboring some types of mutations) of selection during standard virus evolution. In this manner, a high rate of oligonucleotide elongation translated into undetectable mutant frequency outside the bottleneck regimen ([22]; reviews in [5,23]). This example shows that calculation of a true mutation rate either for a specific mutation or as the average for a set of mutations or nucleotide positions is more difficult than the calculation of a mutation frequency.

Nuances of mutation rate calculations apply both to viruses and cells, including bacteria. Problems comprise (i) the use of different units (mutations per nucleotide versus mutations per genome), (ii) not considering the mode of viral genome replication in terms of template utilization (multiple copies produced from the same template versus each progeny molecule becoming a new template; probably an intermediate situation occurs in most viruses), and (iii) bias caused by selection intervening between the biochemical event that defines the mutation rate and the actual mutant quantification to give the mutation frequency [24,25,26,27,28,29,30,31,32].

Concerning (i), it is worth mentioning that the use of mutations per nucleotide as a unit of mutation rate does not take into consideration that accumulation of mutations in the same genome has a limit, thus generally decreasing the frequency of those genomes harboring multiple mutations. Regarding (ii), the problem of replication mode can become significant for comparative purposes if a virus displays marked asymmetry in template usage by positive or minus (complementary) strand and if the replicative polymerase complex has a different protein composition (that affects fidelity properties) when synthesizing plus or minus strands. In addition to amino acid substitutions in the protein subunit that harbors the catalytic polymerization domain, substitutions at other proteins (from the replication complex or associated with it) can also affect template copying fidelity (examples are the coronavirus nsp10 [33], alphavirus helicase/protease nsP2 [34], FMDV non-structural protein 2C [35], and some proteins of yellow fever virus [36]).

A selection-associated bias takes place when the presence of the mutation whose rate is to be quantified affects the multiplication of the virus or bacterium under study. The first mutation rate for an RNA virus that was calculated was for the direct reversion of an A to G mutation introduced at the 3′ extracistronic region of genomic Qβ RNA, which was part of the methodology that gave birth to reverse genetics [37,38]. The mutation rate was estimated in 10^−4^ mutational events per genome doubling. Both mutation occurrence and competition between the mutant and wild-type phage were considered in the calculation, thus eliminating selection bias. Its limitation was that it referred to only one mutation type at a specific genomic site. 

Selection bias affects quantification of mutation rates based on genotypic and phenotypic markers, including the classic fluctuation test. Statistical methods have been developed for correction of selection bias. One of them is the Ma-Sandri-Sarkar maximum likelihood estimator, available at the Fluctuation Analysis Calculator (FALCOR) [39,40,41]. When no correction for selection bias is applied, the discrepancy between a mutation frequency determined experimentally and the underlying mutation rate will depend on the fitness effect of the mutation. The difference will increase with the number of viral or cellular multiplication rounds elapsed between the initial mutational event and the time of the mutant frequency measurement. 

Some phenotypic transitions used as mutation frequency markers (i.e., resistance to a neutralizing monoclonal antibody or to an inhibitor of viral replication) may depend on more than one mutation, resulting in underestimation of the mutation frequency if assumed to be due to a single point mutation. This, together with fitness effects, probably explains the broad range of values calculated for the frequency of monoclonal antibody-resistant mutants in viral populations (as broad as 10^−3^ to 10^−7^ escape mutants per infectious unit with values that are independent of the serological diversity of the viruses in nature; reviewed in [5]). 

Studies with several viruses and their polymerases suggest that the following structural and environmental factors can influence the actual mutation rate at a genomic nucleotide site:

- The structure of the protein that contains the catalytic domain for nucleotide polymerization because it can directly or indirectly affect the interaction of the catalytic residues (and their neighbors) with template and primer residues, as well as with the incoming nucleotide;
- Other subunits or proteins that functionally interact with the protein that contains the polymerization catalytic site;
- Micro-environment in which template copying takes place (ionic composition, temperature, presence of metabolites);
- The sequence context in the template;
- Presence of functional proofreading-repair activities either as part of the polymerase or in proteins that interact with the polymerase;
- Availability and functionality of post-replicative repair pathways;
- Host-coded editing enzymes that may introduce viral genome mutations, unrelated to attributes of the replication complex.

The above points summarize a (probably minimum) number of influences on mutation rates (overviews in [5,24,42]). Main support for the effect of these influences has come from characterization of fidelity mutants of several RNA viruses [43,44,45,46,47,48,49,50,51,52,53,54,55,56], studies with purified viral RNA-dependent RNA and DNA polymerases in vitro [57,58,59,60], and the recognition of the influence of minor tautomeric forms in mutagenesis [61,62]. 

Considering the several influences and uncertainties that preside mutation rate determinations, it is reassuring that there is a wide consensus in that the average mutation rates for RNA viruses fall in the range of 10^−3^ to 10^−6^ mutations incorporated per nucleotide copied. The range is one or more orders of magnitude higher than that estimated for replication of cellular DNA under normal metabolic conditions. High mutation rates stand as a general feature of RNA viruses, other RNA genetic elements, and many DNA viruses. They push viruses towards exploring portions of sequence space where mutations and combinations of mutations are tolerated, waiting to be selected when the environment so demands.

## 3. Limits to Mutation Rates: The Need of Repair

The difference in copying fidelity between the replication complexes of viruses and cells (the latter considered globally for replicative DNA polymerases) suggests that mutation rates have been modulated historically by biological requirements. The fact that mutation rates are affected by amino acid substitutions in viral polymerases and also in other viral proteins (Section 2)—together with the evidence of intra-population mutation rate heterogeneity [63]—implies that mutation rates are evolvable [64]. Interpretations of why their values have settled within the observed limits include: (i): they are a compromise to ensure long-term stability of the core genetic information (understood as the one that permits virus classification) and to cope with changing environments, in particular following bottleneck events that reduce genome diversity; (ii) they are the result of a trade-off between speed of RNA synthesis—assumed to confer a selective advantage to viruses—and copying fidelity; and (iii) they are a requirement of viral dynamics during infection of individual hosts—invasion of different intra-host compartments—and transmissibility [5,11,12,23,65,66]. 

These models are interconnected and not mutually exclusive. Bottleneck events of different intensity (given by the number of founder genomes) are particularly noteworthy because of their abundance in the course of virus life cycles. They occur within infected hosts when viruses transit from one compartment to another and during host-to-host transmission. Bottlenecks permit genomes to explore new regions of sequence space, facilitated by newly arising genome heterogeneity (see as examples [65,66]).

From the available evidence, we consider it unlikely that a specific trade-off—such as speed of RNA synthesis, energy cost of repair (see Section 5), or other—could impose high mutation rates if the latter were not in compliance with long-term core information stability and adaptability requirements. Mutants displaying higher or lower fidelity than their parental wild-type viruses (Section 2) are often attenuated, show defects in replication, and are not found as dominant genomes in mutant spectra evolving in nature. There are biochemical and evolutionary arguments that favor the view that primitive replicons displayed high error rates [67,68]. It is tempting to propose that error-prone replication is not a biological novelty of the cellular-viral world. Rather, it appears to be an inheritance of an ancient RNA (or RNA-like) world. The true novelty seems to be the development of error-correction mechanisms. 

Mutation rates in the range of those observed with RNA viruses would not allow survival of complex genomes (complex in terms of amount of genetic information that they carry). John Drake calculated mutation rates for some DNA viruses and cellular microbes [69,70,71]. He obtained evidence that DNA viruses and organisms mutate at an approximately constant rate of 0.003 mutations per genome (note the units) and multiplication round [71], an observation known as Drake’s rule. It suggests a limitation of the mutation load tolerated by complex genomes, expected from fitness losses of genomes with multiple mutations, with the exception of constellations of either tolerated combinations of mutations or of positive epistasis (fitness gain by the presence of two or more mutations in the same genome as compared with their effect when present individually). In line with genome size-dependent mutation intolerance, a reduction in the bacterial genome size resulted in an increase in mutation frequency [72].

Limitation of mutation acceptability by complex genomes is recapitulated in the error threshold relationship of quasispecies theory. This relationship sets a limit to the maximum permissible error rate, depending on the complexity of the information to be maintained. More genetic information—embodied in large genomes—implies vulnerability to mutations [73,74,75,76]. Terminal (irreversible) deterioration of the genetic information due to mutations is known as entry into error catastrophe. Molecularly it can be visualized as a transition (sometimes referred to as “melting”) from a quasispecies distribution into random sequences (without biological meaning). To avoid error catastrophe, replicative systems have developed proofreading repair (occurring at the replication forks of polymerase complexes) and post-replicative repair activities (operating on finalized error copies once they have been synthesized). Types of DNA lesions that can be corrected by these mechanisms include base substitutions, single and double strand breaks, DNA adducts, DNA-protein crosslinks, and other types of double helix perturbations [77,78]. There are several lines of evidence that suggest that the achieved preservation of genome integrity may protect from accelerated aging and cellular transformation (cancer development). The importance of error correction is also suggested by the more than 100 proteins encoded by the human genome that are devoted to repair functions [78]. Post-replicative repair activities are essential for life stability. They operate efficiently only on double stranded DNA but not on double stranded RNA or DNA-RNA hybrids. Therefore, the cellular post-replicative repair functions that have been characterized to date are ineffective for RNA virus replication products whose error input limitation has to rely on proofreading repair at the step of genome synthesis [77,78,79]. 

## 4. Repair Mechanisms in Viruses

Viruses are no exception to the need of repair functions, as evidenced by the presence of 3′ to 5′ exonuclease (Exo N) activities in complex bacterial and animal DNA viruses and some RNA viruses. Several decades of study have established the role of such activities in DNA copying fidelity, in determining mutator phenotypes when they have been inactivated, and at least in some cases, in promoting DNA recombination ([80,81,82,83,84]; see also other articles of the same *Virus Research* issue [84], among many studies). The extent of functional interdependence between polymerase and Exo N activities varies depending on the virus. In vaccinia virus, the two activities appear to be coupled [84], while in herpes simplex virus 1, mutations in the exonuclease do not necessarily impair the polymerase activity [85]. It appears to be a subtle relationship, as also suggested by studies with coronaviruses (Section 5).

**Figure 2:**
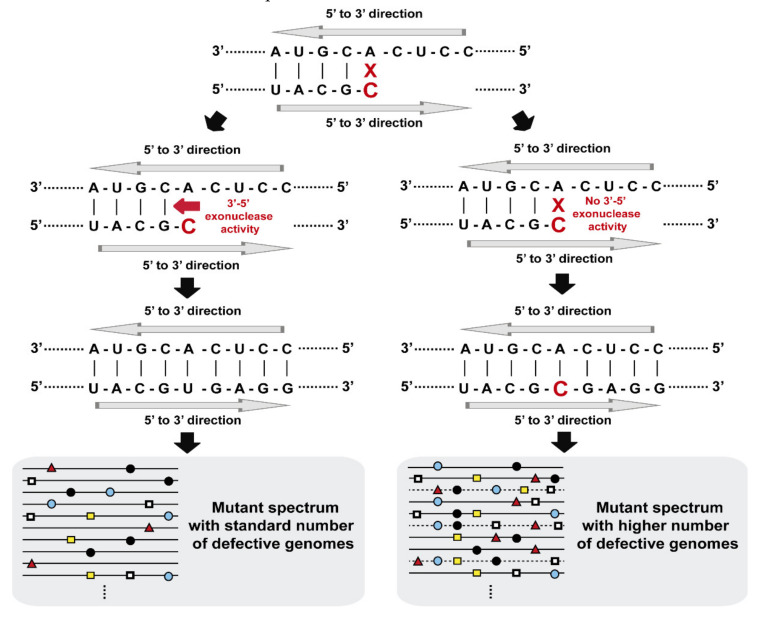
Scheme that illustrates mutant spectrum complexities in the absence and presence of a 3′-5′ exonuclease activity during viral replication. Residue A (on the top) can be misread to incorporate a C in the complementary strand. In the presence of a proofreading activity, the mutant spectrum (represented by lines with mutations as colored symbols) contains a standard number of defective genomes (depicted on the left). In the absence of a proofreading activity, there is an accumulation of mutations per genome that can eliminate viral replication (indicated by discontinuous lines of mutant spectrum on the right).

Repair functions have also been described for RNA viruses. In this case, a distinction can be made between repair activities that compensate specific lesions in primer or terminal sequences relevant to replication functions and those, such as Exo N, that mediate a bona fide reduction in mutation rate (Figure 2). When elongation of IV RNA was attempted with a primer to which 3′ guanosine residues had been added, elongation did not proceed until the excess of GMP residues had been removed [86]. NTP-dependent excision of the 3′ nucleotide at the growing nucleic acid product has been identified for hepatitis C virus and HIV-1 [87,88,89]. A 3′-end repair mechanism has been described for satellite RNAs of some plant viruses [90,91]. Coronaviruses exhibit an Exo N activity that increases template copying fidelity and confers decreased sensitivity to some antiviral agents [92]. Its implication in SARS-CoV-2 biology and diversification has become a focus of interest with the COVID-19 pandemic.

## 5. The Coronavirus Exonuclease Activity, and Additional Considerations on Repair Evolvability

Coronaviruses have the largest single-stranded RNA genomes described to date, reaching up to 32 Kb. They include in their polymerase complex a multifunctional protein (nsp14) with an Exo N domain. Its activity was first confirmed with biochemical assays using recombinant SARS-CoV nsp14 with synthetic RNA substrates. Its presence fits the genome size-dependent limitation of mutation tolerance predicted by the error threshold relationship of quasispecies theory (Section 3). A similar function has not been described for other RNA viruses of smaller genome size. For example, no evidence of a proofreading-repair activity was obtained in the vesicular stomatitis virus polymerase using several biochemical tests [93]. No domain with residues compatible with an Exo N activity has been described in other viral RNA replicases. 

Inactivation of the coronavirus Exo N resulted in impairment of viral RNA synthesis [94,95]. Viable Exo N defective mutants were rescued for murine hepatitis virus (MHV) and SARS-CoV but not (at the time of this writing) for MERS-CoV and SARS-CoV-2 [95]. The SARS-CoV-2 nsp14 displays exonuclease activity in vitro [96,97,98,99] but, to our knowledge, to what extent it lowers the SARS-CoV-2 mutation rate has not been reported. MHV with an inactivated Exo N displayed impaired replication and about a 15-fold higher mutation frequency than the standard virus [92,100,101]; Exo N-defective mutants protected immunocompromised mice from lethal disease [102] and were more susceptible to nucleoside analogues (including lethal mutagens) than standard virus [103,104]. The mutants gained fitness upon passage in cell culture without reversion of the Exo N-inactivating mutations [104].

SARS-CoV-2 protein nsp14 is multifunctional in that it contains both the Exo N and a guanine-N7-methyltransferase (N7-MTase) domain. Removal of the N7-MTase domain maintains the Exo N fold and its exoribonuclease activity, as in the entire nsp10 (polymerase)-nsp14 complex [97]. The coronavirus Exo N promotes genetic recombination [105]. Some studies have associated amino acid substitutions in nsp14 with higher diversity and increased rate of SARS-CoV-2 evolution. However, this provides only indirect evidence of its possible role in replication fidelity, and quantifications based on genetic or biochemical methods are needed. Comparisons between proofreading-repair competent and incompetent SARS-CoV-2 in model diversification tests would be highly revealing. As COVID-19 continues to affect the human population, an increasing number of SARS-CoV-2 variants exhibit high epidemiological fitness. In addition to single nucleotide mutations, insertions, and deletions—very abundant among SARS-CoV-2 isolates from patients, as compared with other pathogenic RNA viruses—may also be favored by Exo N as part of its recombination-promoting activity [21,105].

A theoretical study evaluated the balance between costs and benefits of proofreading in coronaviruses, considering as main variables the effects on template-copying accuracy (to ensure sufficient viable progeny), the speed of RNA synthesis, and energy costs of repair (i.e., ATP molecules consumed and the required enzymatic machinery) [106]. The results suggest that coronavirus proofreading may be beneficial to maintain a sufficient number of virus functional copies but with limits imposed by proofreading costs. Based on the accumulated theoretical and experimental results, we conjecture that the RNA genome complexity increase represented by a transition from around 20 Kb to 30 Kb coding capacity (open-reading frames and regulatory regions) may be critical for the requirement of some error-correction activity. The sharpness of this difference is also supported by the fact that hitherto no RNA viruses within the genome size range of 3 Kb to 20 Kb include a bona fide misincorporation-correcting function. If it were necessary for information stability, the evolutionary requirements for the incorporation of a repair function would not represent an unsurmountable barrier. For example, the laboratory adaptation of an Archeal DNA polymerase (*pol B*) generated an enzyme variant enriched with reverse transcriptase (RT) activity with a proofreading function that lowered the error rate 3- to 10-fold relative to that measured with the RT of Moloney murine leukemia virus [107]. This study suggests that if proofreading activities had been a necessity to counteract an undesired high mutation rate (for example imposed by the RNA synthesis speed) they would be present among the many retro-transcribing entities that lack them. 

It is not clear how an active versus an inactive proofreading-repair activity might impact coronavirus genome diversity at the epidemiological level. It is very unlikely that the question could ever be empirically answered. Suggestions may be ventured from estimates of the number of cells susceptible to SARS-CoV-2 in the human respiratory tract (and other tissues and organs [108]), the number of particles and infectious units in each infected cell, the viral yield per cell, the half-life of the virus, the total viral production in a typical human infection (a wide range of 3 × 10^9^ to 3 × 10^12^, with a range for infectious particles of 3 × 10^5^ to 3 × 10^8^), and the corresponding total number of viral particles circulating at a given time of the COVID-19 pandemic [109]. With these estimates, a 15-fold increase in fidelity (if that were the consequence of the SARS-CoV-2 Exo N activity based on results with other coronaviruses) would imply a comparable exploration of sequence space only after a few more rounds of infection, or a modestly higher number of infected individuals. The advantage of an active proofreading-repair in sustaining genome viability may not affect substantially the long-term genetic and antigenic diversification of the virus, whose progression may threaten vaccine efficacy [21,110,111,112,113,114]. In the context of the explosive COVID-19 pandemic, the rate of evolution of SARS-CoV-2 (inter-host temporal variation of consensus sequences is (1.2 ± 0.5) × 10^−3^ mutations per site and year (with a narrow range of 9.9 × 10^−4^ to 2.2 × 10^−3^ mutations per site and year; this is the average of ten independent measurements reported by other authors; calculation by L. Vázquez-Sirvent, B. Martínez-González, M.E. Soria, and C. Perales, unpublished results). This rate is comparable with that calculated for other RNA viruses under active viral transmission. Important pending questions are the extent to which the SARS-CoV-2 Exo N decreases the error rate of the virus in vivo, and whether phenotypic diversification will represent a problem for COVID-19 control, as it does for other RNA viral diseases [5,114].

## 6. Summary, Conclusions, and Further Comments

The range of mutation rates and frequencies exhibited by RNA viruses, with or without a functional proofreading-repair activity in their replication complexes, is compatible with the rapid development of mutant spectra that are an asset for adaptability. So great is the tendency towards diversification that, at least according to studies with HCV, long-term, monotonous replication in a cell culture environment does not deter the system from continuous exploration of sequence space via expansion of the mutant spectrum [115,116,117,118]. An unknown in viral population dynamics is the time required for a set of infected cells to produce a sufficiently diverse population of infectious particles so as to confer adaptive value to the mutant swarm. Particularly relevant is the relationship between that time and the time it takes for environmental fluctuations to impact the viral population. Although viruses are indeed efficient in adapting, the time span between stimulus and response may mark differences in the scope of adaptability.

In the present review, based on theoretical and experimental studies on the stability of genetic information, we have suggested that a proofreading-repair function of the type described for coronaviruses may have a positive impact on production of a sufficient number of viable genomes to sustain the infection in host cells and organisms. However, it appears less likely that the presence of a repair activity has a significant influence on virus diversification in an epidemiological context. 

Either with repair activity or not, some important problems for disease control that arise from viral population dynamics are likely to apply to many viral pathogens. One is a sustained population disequilibrium even when a virus replicates extensively in an invariant biological environment (reviewed in [118]). The second problem is that limited attention has been paid to mutant spectrum dynamics in comparison with temporal variations of consensus sequences. This would be a minor omission if the analysis of consensus sequences had epidemiological studies as its main focus and a survey of circulating antigenic types to update the composition of antiviral vaccines. The problem arises when the information in data banks is used to prepare universal vaccines or antiviral agents. Efforts along these lines are ongoing for highly variable RNA viruses, including attempts to design pan-coronavirus vaccines. If our results with HCV apply to other variable pathogens, the degree of residue conservation in mutant spectra is far less strict than conservation as inferred from sequence alignments in data banks [119,120]. Since the target of antiviral agents are mutant spectra rather than consensus sequences (which embody limited biological information [12]), prospects of success of universal ligands or immunological stimulants such as vaccines are contingent upon minority variants remaining at low frequency due to their limited fitness. When fitness-enhancing pathways are available, universal ligands are unlikely to impede selection of escape mutants. For this reason, we have proposed expansions of sequence information in data banks by including mutant spectra sequences in addition to consensus sequences [120]. The proposal appears feasible, given the available computer power and big data methodology.

In summary, the potential of many viral pathogens to vary genetically and antigenically remains an important challenge for disease control, but means are now available to try to limit the selection of treatment-resistant viral mutants.
